# The Interplay Between Neuroinfections, the Immune System and Neurological Disorders: A Focus on Africa

**DOI:** 10.3389/fimmu.2021.803475

**Published:** 2022-01-13

**Authors:** Leonard Ngarka, Joseph Nelson Siewe Fodjo, Esraa Aly, Willias Masocha, Alfred K. Njamnshi

**Affiliations:** ^1^ Brain Research Africa Initiative (BRAIN), Yaoundé, Cameroon; ^2^ Neuroscience Lab, Faculty of Medicine & Biomedical Sciences, The University of Yaoundé I, Yaoundé, Cameroon; ^3^ Department of Neurology, Yaoundé Central Hospital, Yaoundé, Cameroon; ^4^ Global Health Institute, University of Antwerp, Antwerp, Belgium; ^5^ Department of Pharmacology and Therapeutics, Faculty of Pharmacy, Kuwait University, Safat, Kuwait

**Keywords:** neuroinfection, neurological disorder, immune system, neuroinflammation, sub-Saharan Africa, neuropathy, pathogen, central nervous system

## Abstract

Neurological disorders related to neuroinfections are highly prevalent in Sub-Saharan Africa (SSA), constituting a major cause of disability and economic burden for patients and society. These include epilepsy, dementia, motor neuron diseases, headache disorders, sleep disorders, and peripheral neuropathy. The highest prevalence of human immunodeficiency virus (HIV) is in SSA. Consequently, there is a high prevalence of neurological disorders associated with HIV infection such as HIV-associated neurocognitive disorders, motor disorders, chronic headaches, and peripheral neuropathy in the region. The pathogenesis of these neurological disorders involves the direct role of the virus, some antiretroviral treatments, and the dysregulated immune system. Furthermore, the high prevalence of epilepsy in SSA (mainly due to perinatal causes) is exacerbated by infections such as toxoplasmosis, neurocysticercosis, onchocerciasis, malaria, bacterial meningitis, tuberculosis, and the immune reactions they elicit. Sleep disorders are another common problem in the region and have been associated with infectious diseases such as human African trypanosomiasis and HIV and involve the activation of the immune system. While most headache disorders are due to benign primary headaches, some secondary headaches are caused by infections (meningitis, encephalitis, brain abscess). HIV and neurosyphilis, both common in SSA, can trigger long-standing immune activation in the central nervous system (CNS) potentially resulting in dementia. Despite the progress achieved in preventing diseases from the poliovirus and retroviruses, these microbes may cause motor neuron diseases in SSA. The immune mechanisms involved in these neurological disorders include increased cytokine levels, immune cells infiltration into the CNS, and autoantibodies. This review focuses on the major neurological disorders relevant to Africa and neuroinfections highly prevalent in SSA, describes the interplay between neuroinfections, immune system, neuroinflammation, and neurological disorders, and how understanding this can be exploited for the development of novel diagnostics and therapeutics for improved patient care.

## 1 Introduction

### 1.1 The Burden of Neurological Diseases and Neuroinfections in Africa

**Figure 1:**
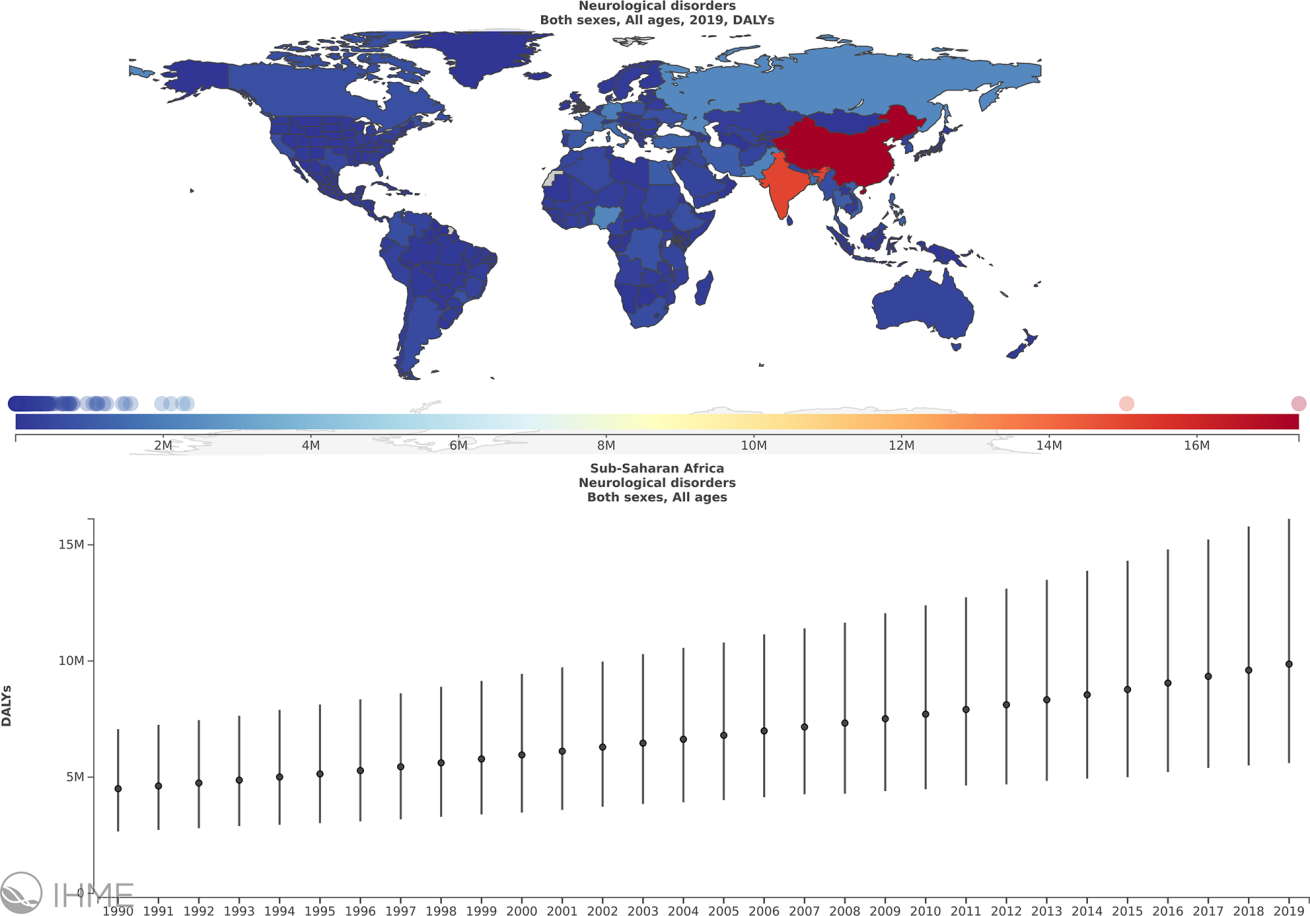
The burden of neurological disorders in sub-Saharan Africa (SSA) over the period of 1990 to 2019. Annual % change in DALYs/100000, Global, Both sexes, All ages, Neurological disorders, Sub Saharan Africa. Obtained from (2) with permission.

Neurological disorders represent a major cause of disability for patients and an economic burden globally. In 2016, neurological disorders, comprising 11.6% of global disability-adjusted life-years (DALYs), were ranked as the leading cause of DALYs and the second leading cause of death (16.5% of total global deaths), after cardiovascular diseases (1). Despite being relatively scarce, the available data suggest that the prevalence of neurological diseases in Sub-Saharan Africa (SSA) has been increasing over time (**Figure 1**) (2) but it is considered lower than other parts of the world (3). Nevertheless, according to the World Health Organization (WHO)-World Federation of Neurology joint report, the burden of neurological disease is underestimated by traditional methods of assessment. The burden is further increased in low-income countries, especially in Africa, because of insufficient human and infrastructural resources, coupled with systems unpreparedness to detect and manage these conditions (4). The overall point prevalence of neurological disorders reported in studies carried out in various hospitals in countries of the SSA region was 3.3% in Uganda (3), 4.2% in Nigeria (5), 7.5% in Kenya (6), 8.5% in Tanzania, 2009 (7), and 10% in Zambia (8). However, some retrospective studies reported higher percentages of neurological disorders in patients admitted to some hospitals; 15% in a hospital in Ghana (Sarfo et al., 2016) and 18% and 24.7% in two tertiary hospitals in Ethiopia (9). The most frequently reported neurological disorders were peripheral neuropathy, chronic headaches, epilepsy, pain syndromes, stroke, and tremors/Parkinson’s disease (3, 7). In SSA, neuroinfections contribute significantly to the diagnosed neurological disorders (10–12), in some cases constituting 26.7% to 43% (5, 13). These neuroinfections include human immunodeficiency virus (HIV), tuberculosis, meningitis, cerebral malaria, rabies, and tetanus (4–7, 9, 10, 13).

### 1.2 Inflammation, Neuroinfections and Neurological Disorders

Neuroinfections result in neuroinflammation, which involves immune cell infiltration into the central nervous system (CNS) from the periphery, chronic astrocyte, and microglia activation, increased chemokine, and cytokine expression, to control or eliminate the pathogens but can also be detrimental to the host (14–17). The interplay between infectious pathogens and the immune system in the CNS is covered in more detail in various articles in this Research Theme (Neuroimmunology in Africa). The pathogenesis of neurological disorders is associated with neuroinflammation in general or due to infections (14, 17–20). Neuroinflammation caused by HIV, tuberculosis, cerebral malaria, neurocysticercosis, cerebral toxoplasmosis contributes to the pathogenesis of epilepsy that occurs during or after these infections (21–24). Alteration of the immune system caused by HIV contributes to the pathogenesis of neuropathy (25, 26). Similarly, a possible pathogenic mechanism of sleep disturbances observed in human African trypanosomiasis (HAT) patients is the upregulations of cytokines such as interleukin-1 beta (IL-1β) and tumor necrosis factor-alpha (TNF-α) (17, 20, 24).

This review addresses the interplay of the immune system and neuroinfections in the pathogenesis of certain neurological diseases prevalent to the SSA region such as epilepsy, dementia, motor disorders, headache, sleep disorders, and peripheral neuropathy.

## 2 Specific Neurological Disturbances Related to Neuroinflammation and Brain Infections

### 2.1 Epilepsy

#### 2.1.1 Introduction

Epilepsy is a chronic disease of the brain that affects an estimated 50 million people worldwide according to the WHO (27). It manifests as repetitive, involuntary epileptic seizures that vary in their clinical presentation (28). The overall lifetime prevalence of epilepsy is estimated at 7.60 per 1,000 population (29). About 80% of the global burden of epilepsy occurs in individuals residing in low and middle-income countries (LMICs) (27). In SSA specifically, a median epilepsy prevalence of 14.2 per 1,000 was documented; over 90% of the patients were aged below 20 (30). Annual epilepsy incidence was also high, reaching 81.7 per 100,000. Mortality was greatest in the 18-24 years age group, suggesting a relatively low life expectancy among persons with epilepsy (PWE) in Africa (30). The main risk factors for epilepsy reported in resource-poor settings include perinatal brain insults, traumatic head injury, and infections of the CNS (11, 30).

#### 2.1.2 Physiopathology of Epilepsy and Common Infectious Etiologies

**Figure 2:**
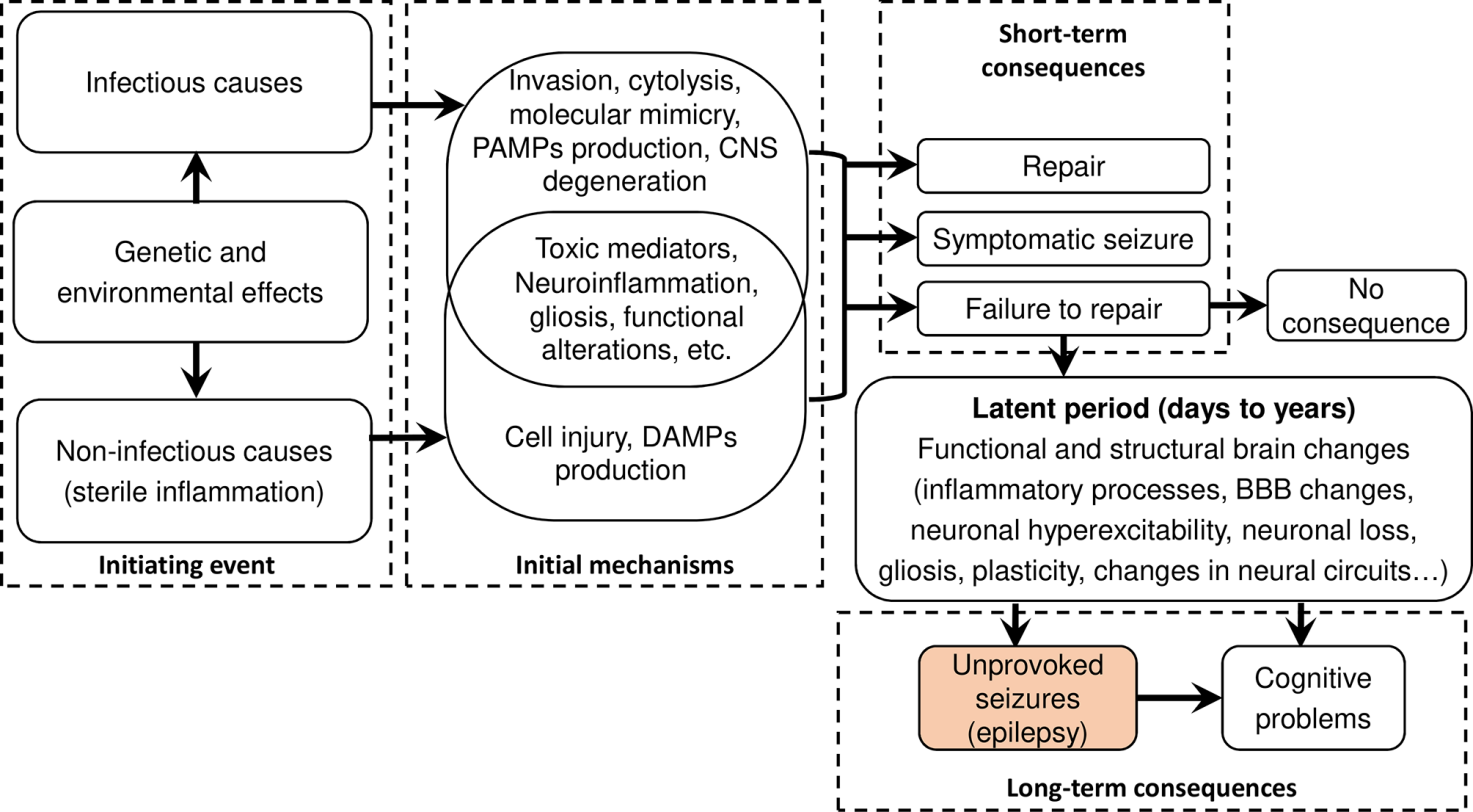
Flow diagram summarizing infectious and sterile epileptogenic mechanisms. After an initial brain insult, a cascade of processes occurs that, under certain circumstances, could create an enduring predisposition to unprovoked seizures. Both damage-associated molecular patterns (DAMPs) in sterile infection and pathogen-associated molecular patterns (PAMPs) activate pattern recognition receptors (PRR) leading to overlapping pathways for inflammation and long-term pathological consequences. Figure adapted from (24).

Epilepsy is characterized by an enduring predisposition to generate epileptic seizures (28). Diverse mechanisms underpin epileptogenesis and are often a consequence of brain insults and the resulting inflammation (24). The International League Against Epilepsy (ILAE) recently highlighted six main categories as etiologies for epilepsy: structural, genetic, infectious, metabolic, immune, and unknown etiologies (31). The interplay between brain infections and inflammation, and how these may lead to epilepsy are summarized in **Figure 2**. During an initial brain insult, proinflammatory cytokines (principally IL-Iβ, IL-2, and IL-6) produced by glial cells and neurons may cause cerebral damage (32). Of note, the released cytokines also activate astrocytes and microglia leading to increased production of cytokines by the latter, thus creating a vicious circle. Furthermore, proinflammatory molecules may reach the CNS hematogenously during disseminated systemic inflammation, particularly when the blood-brain barrier (BBB) is compromised (33). Incomplete tissue repair following an initial brain insult could result, after a certain latent period, in a permanent seizure-causing lesion. Between the time of the initial lesion and the development of epilepsy (latent period), several processes occur including brain neuronal hyperexcitability facilitated by both N-methyl-D-aspartate (NMDA) receptor and other glutamate-mediated mechanisms, neuronal loss and gliosis, molecular and structural reorganization, and epigenetic reprogramming; all these processes may ultimately result in recurrent unprovoked epileptic seizures (24).

Although epilepsy can result from non-infectious causes such as traumatic brain injury, hypo-anoxic episodes, or metabolic anomalies, the most common preventable causes of epilepsy in SSA are infections that affect the CNS (22, 30, 34). Epilepsy from an infectious etiology should be understood, as the unprovoked seizures that persist even after the resolution of the acute infection (31). The proportion of epilepsies attributed to infection varies widely from one study to another, ranging from 1% to 47% (30). Infectious etiologies include neurocysticercosis, tuberculosis, HIV, cerebral malaria, subacute sclerosing panencephalitis, cerebral toxoplasmosis, and congenital infections such as Zika virus and cytomegalovirus (31, 35). Recent cohort studies from Cameroon support the addition of onchocerciasis to this list of etiologies of infectious epilepsy, as children with higher *Onchocerca volvulus* parasitic loads had an increased risk of developing epilepsy later in life (36, 37). These CNS infections can cause a structural cerebral lesion which will act as an organic basis for seizure recurrence even after anti-infectious treatment; therefore substantial overlap exists between infectious and acquired structural causes of epilepsy (38). For instance, neurocysticercosis, perilesional inflammation around the space-occupying lesions may lead to epileptogenesis either by causing gliosis and/or BBB dysfunction (39). Common CNS infections known to cause epilepsy are summarized in **Table 1**.

**Table 1:** Infections of the central nervous system implicated in epilepsy (23, 24, 40).

Infectious agents	Mechanism(s)	Clinical consequences
**Viruses**: arboviruses, coxsackie, enterovirus, rubella, measles, HIV, herpes simplex, cytomegalovirus, flavivirus (Japanese encephalitis), Dengue	- CNS invasion/Inflammation/release of cytotoxic substances/increased neuronal excitability/necrosis - Secondary infections of CNS & metabolic disorders in HIV infection.	Meningitis/encephalitis/encephalomyelitis, epilepsy
**Bacteria**: Meningococcus, pneumococcus, *Haemophilus influenzae B* (Hib), *Mycobacterium tuberculosis*	CNS invasion/Inflammation/intracerebral lesions	Meningitis/cerebral abscesses/intracranial empyemas, epilepsy
**Parasites:** *Taenia solium, Plasmodium falciparum, Naegleria fowleri, Entamoeba histolytica, Trypanosoma spp, Onchocerca volvulus, Toxocara canis, Echinococcus granulosus, Toxoplasma gondii*	- CNS invasion/Inflammation/encephalitis/intracerebral lesions/autoimmunity? - Combination of parasites increases epilepsy risk (41)	Cerebral abscesses/cysts/calcifications, epilepsy
**Fungi:** *Cryptococcus neoformans, C. immitis, H. capsulatum, Candida albicans, A. fumigatus, A. flavus, Mucoraceae* sp., Aspergillus, Blastomyces, Histoplasma	CNS invasion/Inflammation (immunocompromised++)	Meningitis/abscesses, vasculitis/capillary thrombosis, epilepsy

#### 2.1.3 Diagnostic and Management Approaches for Epilepsy in SSA

The diagnosis of epilepsy is essentially clinical and based on criteria set by the ILAE. Practically, epilepsy can be considered as two unprovoked (or reflex) seizures occurring >24 h apart, or one unprovoked seizure and a probability of at least 60% for spontaneous seizure recurrence in the next 10 years (42). Paraclinical investigations to diagnose epilepsy include: electroencephalography (EEG), brain imaging (by computed tomography [CT] scan or magnetic resonance imaging), and blood analysis to exclude metabolic, genetic, or autoimmune causes. Since all paraclinical investigations are not always feasible in some SSA settings, the clinical characteristics of seizures may orientate towards specific etiologies and guide management. For instance, focal seizures in persons with epilepsy could be symptomatic of neurocysticercosis, particularly if accompanied by other focal neurological deficits (43).

The main objective of epilepsy treatment is seizure control. This can be achieved using anti-seizure medications, which must be taken daily. The anti-seizure medications routinely used in LMICs and recommended by the WHO include phenobarbital, carbamazepine, phenytoin, and valproate (44). Regarding preventive management, public health interventions should be instituted to avert epilepsy of infectious origin in SSA. Firstly, institute strategies to prevent any initial insult to the brain and control the incriminated infective agents. The public health actions to fight against neurocysticercosis and onchocerciasis in endemic foci constitute a good example of preventing epilepsy caused by those infections (45, 46). Secondly, treating the cause of an eventual CNS infection can modify the prognosis of the disease. However, epilepsy results from an enduring epileptogenic state, thus treating the underlying infection – for epilepsy of infectious origin – may not completely reverse epileptogenicity. Nonetheless, anti-infectious treatment could reduce the infectious load and improve epilepsy outcomes. The *O. volvulus* parasitic load in persons with onchocerciasis-associated epilepsy correlated positively with seizure frequency and disease severity, and treatment with ivermectin improved seizure outcomes (47, 48). In the same manner, neurocysticercosis cases that received appropriate treatment had higher hippocampal volumes than their untreated counterparts, suggesting more severe brain damage in the latter (49).

#### 2.1.4 Research Gaps and Perspectives

The fact that the very definition of epilepsy depends on seizure recurrence poses a diagnostic challenge. Predicting seizure recurrence after an initial brain insult is still a subject of scientific debate (50). Some progress has already been achieved in this light, as there are now suggested indicators to identify neurocysticercosis patients who are likely to develop epilepsy: those with a strong serologic response (4 bands to *Taenia solium* antigen on neurocysticercosis enzyme-linked immunoelectrotransfer blot (EITB) (39). More research is warranted to understand the infectious threshold and other predisposing conditions that could trigger the development of epilepsy following a CNS infection. Finally, the varied yet understudied epilepsy etiologies in SSA require the establishment of state-of-the-art brain research institutes in the continent and the initiation of North-South collaborations to generate data relevant for the African population.

### 2.2 Dementia

#### 2.2.1 Introduction

Dementia is a neurodegenerative disorder that leads to a progressive deterioration of cognitive functions. It is characterized by a gradual cognitive decline that interferes with independent daily functioning (51). The *Diagnostic and Statistical Manual of Mental Disorders V* (DSM-V) proposes to replace dementia with the term “major neurocognitive disorder” to encompass the wide spectrum of symptoms experienced by the affected persons (52). According to the WHO, Alzheimer’s Disease (AD) is the most common cause of dementia, responsible for 60-70% of all cases (53).

Recent estimates suggest that 0.7% of the world’s population has dementia, translating to about 51.6 to 55 million people worldwide (53, 54). Although nearly 60% of dementia patients live in LMICs, Africa has the least burden of dementia compared to other continents possibly due to its relatively younger population (53, 54). Studies conducted in different populations and geographical regions consistently support that advanced age is a major risk factor for developing dementia (55, 56); indeed, the prevalence of dementia is 2% in those aged 65-69 years, much lower than the 20% in those aged 85-89 (55). Other risk factors for dementia include female gender, low education, cigarette smoking, excessive alcohol intake, diabetes, and hyperlipidemia (55). A meta-analysis of dementia studies in SSA estimated a pooled prevalence of 5.0% for all ages and a pooled annual incidence of 2.0% (56). The number of persons with dementia is expected to rise globally, with the highest increase in prevalence projected to occur in eastern SSA by the year 2050 (57).

#### 2.2.2 Physiopathology and Etiologies of Dementia Secondary to Neuroinfections

During neuroinfection, the initial pathogenic invasion of the CNS induces a diffuse inflammatory process that alters neuronal function (58). The activated microglia release cytokines (IL-1, IL-6, and TNF-α) and neurotoxic agents that further exacerbate CNS damage (58, 59). In chronic neuroinflammation lasting weeks to months, as in the case of some subacute or chronic infections, microglia activation can persist for extended periods, releasing quantities of cytokines and neurotoxic molecules that contribute to long-term neurodegeneration (60). Infections of the CNS associated with dementia (or at least, cognitive impairment) in SSA include HIV, neurosyphilis, and meningitis or encephalitis caused by bacteria, viruses, parasites, or fungi (55). Several infections may occur in the same individual (e.g., neurosyphilis in a person infected with HIV), and it is common to have dementia with mixed infectious and non-infectious etiologies potentiating each other. The “seeding” hypothesis describes a possible mechanism of AD where amyloid-β agglutinate into plaques in a bid to trap a microbe (61). Microbes invade the CNS and stimulate microglia to induce an immune reaction that boosts the levels of an enzyme that helps to produce amyloid proteins. The amyloid protein is meant to act as a defense mechanism, engulfing and disabling the microbes. However, failure to clear these amyloid proteins ramps up inflammation, and eventually amyloid accumulation that constitutes a hallmark for the development of AD (61).

##### 2.2.2.1 Human Immunodeficiency Virus and Dementia

A large proportion of people living with HIV/acquired immunodeficiency syndrome (AIDS) (PLWHA) develop HIV-associated neurocognitive disorders (HAND), independent of opportunistic conditions. A meta-analysis estimated that 45.2% of adult PLWHA in SSA suffer from HAND (62). The pathophysiological mechanisms of HAND are not yet completely understood. Before the widespread introduction of antiretroviral treatment (ART), HIV-associated neuropathology was thought to result from CNS inflammation due to direct penetration and replication of the virus within the microglia and macrophages, as well as neurotoxicity caused by HIV proteins and/or factors secreted from the infected CNS cells (63). However, the persistence of HAND during the post-ART era when several PLWHA have achieved viral suppression warrants additional explanations to the development of neurocognitive symptoms in HIV/AIDS (64). Based on recent research, neurodegeneration in PLWHA most likely involves HIV proteins such as glycoprotein (gp)120, tat, and nef that activate neuroinflammatory and apoptotic pathways, promote oxidative stress, deplete neurotrophic factors, and cause vascular damage (65).

Despite some studies dating back to the 1980s and 90s suggesting that HIV can infect neurons, it appears that the main HIV pathogenic pathway in the CNS is by stimulating infected microglia and macrophages to produce inflammatory factors and reactive oxygen species (ROS) (66). The pathogenesis of HAND entails the following processes (67): (i) CNS tropism by HIV, which causes the viral particles to preferentially invade the brain and spinal cord; (ii) CNS penetration by crossing the BBB mainly *via* adsorptive endocytic mechanisms; (iii) HIV internalization by monocyte/macrophages, which upon crossing the BBB, leads to infection and activation of resident microglial cells by shedding HIV envelope protein gp120; (iv) propagation of infection among microglia accompanied by the release of neurotoxic agents by the latter (TNF-α, IL-1β, glutamate, quinolinic acid) leading to neuronal damage and cognitive dysfunction. Activated microglia also induce astrocyte differentiation and apoptosis and can interfere with normal neurogenesis (68). Interestingly, it has been reported that some HIV proteins can act on the BBB to reduce the entry of antiretroviral drugs (ARVs) (69), thereby making viral suppression difficult in the brain and maintaining the CNS as a potential reservoir for HIV.

Glial cells infected by HIV are involved in inflammatory processes *via* the release of HIV proteins (gp120, Tat, and Vpr) alongside inflammatory cytokines and neurotoxins (66). The released HIV proteins damage neurons and astrocytes (70) and activate virus replication. Secreted cytokines such as IL-1β, TNF-α, and interferon-gamma (IFN-γ) can stimulate viral replication in latently infected glial cells (71), thereby maintaining the CNS infection.

Clinically, HAND patients present with psychomotor slowness, depression, impaired memory, poor visuospatial skills, and impaired executive functions. These symptoms impact the quality of life of the affected individuals. The WHO (72), and the American Academy of Neurology AIDS taskforce (73) clinically classified HAND. The latter classification was recently reviewed (74); the updated version is the most universally used nosology and is considered the gold standard in HIV research; commonly referred to as the *Frascati criteria* for HIV-associated neurocognitive disorders. The Frascati criteria outline three severity levels for HAND: asymptomatic neurocognitive impairment, mild neurocognitive disorder, and the most severe form: HIV-associated dementia (66).

Besides HIV itself, opportunistic neuroinfections in immunocompromised PLWHA can also cause dementia mostly by direct CNS invasion and local brain damage; these include cerebral toxoplasmosis, cryptococcal meningitis, tuberculous meningitis, and cytomegalovirus encephalitis (75).

##### 2.2.2.2 Neurosyphilis and Dementia

CNS invasion by the spirochete *Treponema pallidum*, known as neurosyphilis, is another cause of dementia in Africa. A retrospective study in South Africa found that among 161 patients with neurosyphilis, over half (50.9%) had psychosis/dementia symptoms. Neurosyphilis is responsible for inflammatory processes of the cerebrovascular system and the meninges. Infection with *T. pallidum* may cause chronic meningitis, meningovascular syphilis, or focal gumma in the CNS, which over time may result in dementia (76). The resulting clinical spectrum is wide, ranging from asymptomatic forms to general paralysis which is the most severe presentation of neurosyphilis (also known as dementia paralytica, a condition involving treponemal infection of the brain parenchyma that often presents with cognitive decline and neuropsychiatric symptoms) (75, 77).

##### 2.2.2.3 Other Infectious Causes of Dementia

Encephalitis caused by neurotropic pathogens (herpes viruses; *Borrelia burgdorferi* that causes Lyme disease; hepatitis C; *T. solium* that causes neurocysticercosis may cause cognitive sequelae that could evolve to dementia syndromes in immunocompetent individuals (75, 77). Cerebral malaria is another important cause of cognitive impairment in SSA and a history of it has been associated with long-term mental health disorders and cognitive impairment (78, 79).

#### 2.2.3 Diagnostic and Management Approaches for Dementia in Africa

Diagnosing dementia requires rigorous history taking to document the patient’s daily activities (often requiring the corroboration of the anamnesis by a close friend or family member), in addition to a thorough mental status examination by a clinician to investigate impairments in cognitive functions including memory, language, attention, spatial orientation, executive functions, and mood (80). This may be complemented by a standard battery of neurocognitive and/or neuropsychological tests, brain imaging (by magnetic resonance), and/or investigation of the cerebrospinal fluid (CSF) to investigate the etiology of dementia. Typical CSF findings for some dementias caused by proteinopathies include: reduced amyloid-β, increased tau and P−tau in AD; reduced α−synuclein for Lewy body dementia; real-time quaking-induced conversion, increased 14−3−3 protein, neuron-specific enolase, and tau for Creutzfeldt–Jakob disease (81). Additionally, an infectious workup may be required to rule out common infections associated with dementia as discussed above; this approach seems feasible for resource-limited settings in SSA, where point-of-care tests for HIV and syphilis could be used to raise the index of suspicion regarding a possible infectious etiology when investigating persons with dementia. Persons with dementia of infectious origin who have a positive serology for the infectious disease may also have CSF abnormalities indicating general neuroinflammation (pleocytosis, elevated proteins) or pathogen-specific findings: HIV-ribonucleic acid (RNA), cryptococcal fungi, cytomegalovirus deoxyribonucleic acid (DNA), and a positive Venereal Disease Research Laboratory test for neurosyphilis (75). Finally, genetic testing may be considered in some cases, for instance, those with atypical dementia (81).

Pharmacological treatment of dementia is mainly symptomatic, to improve the patients’ quality of life. Adjuvant therapies, such as anti-inflammatory medications, are relevant for dementia of infectious origin as they downplay the associated neuroinflammation. Limited evidence suggests that early treatment of the infectious etiology may reverse the dementia syndrome altogether, or at least preserve cognitive function; examples in the literature include cases with dementia secondary to some viral encephalitis and neurosyphilis (75).

#### 2.2.4 Research Gaps and Perspectives

Although literature currently reports that Africa is the continent least affected by dementia, the prevalence of dementia in it is expected to rise in the future (57). This could be attributed to the rising life expectancy (82) and the persistence of several neurotropic infections in SSA. Therefore, research capacity should be strengthened to improve novel preventive interventions adapted to the African context, and diagnostic and management capacity for dementias. Indeed, several cases of clinically diagnosed dementia remain uninvestigated due to infrastructural and/or technical limitations in these settings. Finally, Central and peripheral inflammatory pathways could become the targets to prevent the development of dementia among at-risk individuals as peripheral inflammation caused by infections or other causes can exacerbate or trigger central inflammation (83).

### 2.3 Motor Neuron Diseases

#### 2.3.1 Introduction

Motor neuron diseases (MNDs) are a group of neurodegenerative disorders characterized by the selective death of motor neurons. The spectrum of MNDs involves varying degrees of upper and lower motor neuron involvement and is differentiated from neuropathies by the pattern of motor and/or sensory involvement. These disorders range from spinal muscular atrophy (frequent in childhood) to amyotrophic lateral sclerosis (ALS) in adults (84). The most prevalent MND is ALS, which can be inherited or sporadic, and is characterized by mixed upper and lower MND, with sensory sparing (85). The reported all-age global prevalence of MNDs was 4.5 per 100 000 people and all-age incidence was 0.78 per 100,000 person-years, causing 926,090 DALYs and 34,325 deaths in 2016 (86). Africa has an underestimated burden of MNDs as most epidemiological studies were conducted in hospital settings with low prevalence and incidence rates (87, 88). Quansah et al. reported several cases of MNDs in community and hospital settings ranging from 5 to 15/100,000 people and 250 to 750/100,000 people (89). Kengne et al. reported a hospital-based prevalence of ALS of 0.5% in Cameroon (90). Risk factors for MNDs in SSA include severe hypotonia in infants, trauma, family history of MNDs, sensory changes, and spinal anesthesia (82).

#### 2.3.2 Pathophysiological Mechanisms and Infectious Etiologies of MNDs

Motor neuron diseases result from an interplay of genetic, age-related, environmental, and developmental factors (91). The pathophysiological mechanism underlying the etiology, occurrence, and aggravation of MNDs remains not fully elucidated and the center of research. However, recent research using animal models suggests a vital role of glial cells and neuroinflammation in MNDs. Neuroinflammatory processes such as activated microglia, infiltrated T cells, and the subsequent overproduction of proinflammatory cytokines and other neurotoxic or neuroprotective molecules, play a role in the pathophysiology of ALS (92). Several studies propose that the pathophysiology of ALS encompass an exaggerated innate and reduced acquired immunity (93), as well as defective astrocytic clearance of excess glutamate, which results in neuronal excitotoxicity and death (94). These studies provide the basis for further research to understand the role of neuroinflammation in MNDs (95).

Some infections may be able to trigger MNDs, with clinical presentations mimicking ALS. Enteroviruses (a group of positive-stranded RNA viruses including poliovirus, coxsackievirus, echovirus, enterovirus-A71, and enterovirus-D68) have been incriminated in the development of ALS as they can target motor neurons; patients with prior poliomyelitis are at increased risk of developing MNDs (96). Mouse models revealed that infection with enteroviruses induces molecular changes such as defective RNA-processing, impaired nucleocytoplasmic transport, neuroinflammation, compromised protein quality control, and abnormalities of the transactive response DNA binding protein-43 (TDP-43), supporting their involvement in ALS pathogenesis (96). Besides enteroviruses, infection with retroviruses such as HIV has been associated with ALS (97), although further studies are required to firmly establish causality.

##### 2.3.2.1 Motor Neuron Disease Caused by the Poliovirus

The poliovirus is the viral agent responsible for paralytic poliomyelitis, an acute disease of the CNS (specifically the anterior horn of the spinal cord) resulting in flaccid paralysis (98). With the advent of effective vaccines, the number of poliomyelitis cases has been on a steady decline worldwide. The annual incidence of paralytic polio decreased from an estimated 350,000 in 1988 to about 1,000 cases from 2001 to 2004 (99). In addition to the acute disease, the post-polio syndrome is another neuromuscular pathology that affects some poliomyelitis survivors many years after the initial severe disease (100).

The poliovirus is transmitted to man *via* the fecal-oral route. Upon entry into the human host, the poliovirus attaches to host cell surfaces *via* the poliovirus receptor (PVR), a membrane protein (CD155) of the immunoglobulin superfamily. The poliovirus receptor is abundantly expressed in certain tissues such as the nasopharyngeal mucosa, Peyer’s patch M cells of small intestines, the anterior horn motor neurons of the spinal cord, and medulla oblongata (101); this distribution of PVR explains the tropism of the poliovirus for these tissues. Infection and replication of poliovirus result in cell death (apoptosis). Suggested mechanisms of virus-induced apoptosis have previously been reviewed (102–104). In case of poliovirus, apoptosis of neuronal cells most likely involves CD155 and caspases (98). Indeed, poliovirus replication in Tg-CD155 mice models induced DNA fragmentation (characteristic of apoptosis) in the three main CNS cell types (neurons, astrocytes, and oligodendrocytes) (105). Paralysis ensues when a certain threshold of local inflammation and motor neuron death is reached; this happens in less than 1% of infected individuals (99). Recovery from paralysis occurs in only 20-30% of affected subjects, but in the majority, the paralysis is permanent and results in muscle atrophy and joint deformities (101).

##### 2.3.2.2 Motor Neuron Disease Caused by Retroviruses

The human retroviruses (both exogenous and endogenous) have recently been considered as a viral etiology for MNDs. In Tanzania, a 12% prevalence of MND was found among HIV-infected persons, as opposed to only 4.7% in the general population (87). Both the Human T-cell leukemia or T-lymphotropic Virus 1 (HTLV-1) and HIV-1 are implicated in MND neuropathology including the development of ALS-like syndromes (106). The association between ALS and retroviruses was further confirmed through state-of-the-art bioinformatics approaches (107). Several pathophysiological pathways incriminate retroviruses in the development of MNDs. Considering HTLV-1 infection, the exact mechanisms for the neurological disease remain unknown. The main hypothesis to explain HTLV-1-associated myelopathy/tropical spastic paraparesis (HAM/TSP) neuropathogenesis is the so-called “Bystander damage.” It suggests that the presence of IFN-γ-secreting HTLV-1-infected CD4^+^T cells and their recognition by virally specific cytotoxic CD8^+^T cells in the CNS, induce microglia to secrete cytokines, such as TNF-α, which may be toxic for the myelin of neurons. Clinically, HAM/TSP is a slowly progressive neurological condition that is defined clinically and serologically according to the WHO guidelines (108). Alfahad and Nath reported that at least 35 cases of ALS-like syndrome had been documented in literature since the description of HAM/TSP in the 1980s (106).

Dozens of cases with HIV-associated ALS have been documented in the literature (106) but the mechanism is not yet fully understood. Given that evidence suggest that HIV infects infiltrating macrophages, microglia, and astrocytes but not neurons, it is likely that the latter are affected indirectly. In addition to HIV, a human endogenous retrovirus K (HERV-K) in the brain and cortical neurons, which can be activated by the tat protein of HIV, was reported to be a contributor to MND (109, 110). Expression of HERV-K or its envelope protein in neurons placed in culture (*in vitro)* or in experimental animals (*in vivo)* causes motor neuron degeneration producing a similar phenotype to ALS (109). We surmise that possible interactions between HIV and HERV-K can result in MND in PLWHA, and the fact that symptoms regress with ART further supports the role of HIV in the pathogenesis of the motor neuron symptoms (111). It appears that controlling HIV infection using drugs that cross the BBB would indirectly control HERV-K activation in the neurons and result in clinical improvement.

Clinically, PLWHA with MND present with ALS-like symptoms including asymmetric limb weakness, upper and lower motor neuron signs, fasciculations, brisk muscle jerk reflexes, muscle atrophy, and fatigability. However, compared to ALS in the general population, PLWHA have an earlier age of onset of ALS symptoms, rapid progression, and sometimes a favorable evolution when ART is initiated (112).

#### 2.3.3 Diagnosis and Management of MNDs

The classic form of ALS consists of a mixture of upper and lower motor neuron features. ALS patients complain of asymmetrical limb weakness with difficulty handling objects, and decreased muscle bulk; this weakness progressively migrates to other limbs with possible involvement of respiratory muscles (113, 114). The condition is often diagnosed by applying the El Escorial criteria that take into account both clinical and paraclinical elements (electrophysiology and neuroimaging) (115). Recently, potential biomarkers have been suggested to diagnose ALS; the presence of these biomarkers in the blood (e.g.: percentage of monocytes, immunoglobulin M [IgM], and CD3 lymphocyte counts) or CSF (e.g.: Chitinase-3-like protein 1, Chitinase-3-like protein 2, Alpha-1-antichymotrypsin) raises the index of suspicion in favor of ALS, and may discriminate ALS from other MNDs (116, 117). However, more research is needed to establish an appropriate test for ALS. The lack of a definitive test for ALS can be problematic, especially in contexts whereby patients are seen very early when symptoms are still scanty. In these cases, waiting and observation of the disease progression over the next few weeks and months are needed. In patients with paralytic poliomyelitis, the typical clinical picture is that of an MND with generalized weakness followed by asymmetrical flaccid paralysis and conserved sensory functions (118); therefore polymerase chain reaction (PCR) for detection of poliovirus in stool, throat swabs, blood, and CSF may be indicated when confronted with an MND clinical picture (119).

Management of ALS involves a multidisciplinary team to assess pulmonary function, diet, as well as the use of antioxidants and riluzole. As of now, the medications only slow down the course of the disease; there is no cure (114).

#### 2.3.4 Future Perspectives

There has been a wide range of research to elucidate the course and etiologies of MNDs. Unfortunately, most of this research has been done using experimental animals as well as genetic studies to tackle genetic-MNDs. The challenges that however remain are: the scarcity of community-based research of MNDs in Africa as a whole (89), the feasibility of translating these rodent studies to human studies, and the absence of effective drugs to treat or potentially reverse the evolution of MNDs. Failure to meet these challenges to date could be a result of inadequate protocols during rodent studies, including the timing of drug administration, small sample sizes, and differences in the mechanisms of MNDs. Further understanding of the molecular pathology of glial cells will contribute to developing therapies to slow the progression of MNDs and reduce incidence and disabilities (120).

### 2.4 Headache Disorders

#### 2.4.1 Introduction

Headaches are the most prevalent disorders of the nervous system (121). Often underestimated, they usually have an insidious onset. They are divided into primary headaches (such as migraines and tension-type headaches) and secondary headaches, which are a result of an underlying condition (122). Studies have reported a 96% global lifelong prevalence of primary headaches, with females being more affected than males. The active prevalence of tension-type headache worldwide is estimated at 40%, and that of migraine at 10% (123–125). Globally, the prevalence of chronic daily headaches has remained consistent at 3–5% (126), with chronic migraine representing most of it. Headaches are ranked as the second leading cause of years lived with disability (YLD) worldwide with migraine alone accounting for one-third of total YLD in young adults (127, 128). In Africa, recent community-based studies have reported migraine prevalence between 3 to 6.9%, and chronic tension-type headache prevalence at 1.7% (129). In a hospital-based study conducted in Cameroon, headache disorders accounted for about 34% of complaints in out-patient consultation (130).

#### 2.4.2 Immunopathophysiology of Headaches

Since the 1970s, it was suspected that the immune system plays a role in the development of chronic headaches (131). Some key elements in the immune system have been implicated in the pathogenesis of headaches (132). Calcitonin gene-related peptide (CGRP) is an inflammatory neuropeptide that contributes to headache pathophysiology, causes neurogenic inflammation, and activates the peripheral trigeminocervical neuron during the initiation of migraine at the brainstem or cortex level. This induces neurogenic vasodilation, extravasation of plasma proteins, and the influx of mast cells and other proinflammatory cells (133). Based on these initial findings, CGRP-receptor antagonists have been developed to block neurogenic vasodilation in the meninges (134). Other recent studies have revealed that CGRP triggers the secretion of cytokines by stimulating CGRP receptors found on T-cells, resulting in inflammation which might be involved in the pathogenesis of headaches (135).

Plasma levels of both pro- and anti-inflammatory cytokines are enhanced during migraine attacks. The levels of TNF-α increase rapidly and then decrease progressively over time after the onset of a migraine attack (136). Plasma levels of another proinflammatory cytokine, IL-1β, also increase after the initiation of headache. The release of IL-1β is induced by TNF-α and may lead to hyperalgesia. In a small number of patients with new daily persistent headache (NDPH), symptoms may develop after viral infection. In such cases, proinflammatory cytokines such as TNF-α could initiate and maintain CNS inflammation even after the resolution of the infection. Tumor necrosis factor-α is an important component in the pathogenesis of some conditions such as sinusitis and rhinitis, but also in headaches (137). The development of drugs that modulate TNF-α may benefit all these conditions. Adiponectin, which is secreted by the adipose tissue in obesity, is believed to modulate several inflammatory mediators important in migraine. Adiponectin has an anti-inflammatory action through inhibition of IL-6 and TNF-α-induced IL-8 production. Adiponectin also induces the production of cytokine IL-10, which is an anti-inflammatory. Although adiponectin decreases migraine, paradoxically, a sudden increase in its levels may worsen a headache (138). Thus, it is a possible biomarker or therapeutic target for migraine. Another possible immune marker are mast cells; these are granulated immune cells that upon stimulation degranulate and induce a local inflammation. The abundant mast cells in the intracranial dura degranulate their contents into the local milieu, activate the surrounding trigeminal meningeal nociceptors, and promote a prolonged state of excitation (139). The molecules released by mast cells activate the meningeal nociceptors followed by a cascade of neuronal activation mediated by the release of neuropeptides (e.g., CGRP, substance P), which further degranulate residual mast cells and prolong the migraine headache (140).

In secondary headaches caused by infections, the mechanism underpinning the headache symptom is usually non-specific as they largely depend on the causative disease itself and the accompanying inflammation (141). Literature is scarce on the pathogenesis of headaches due to systemic infection; however, the role of fever is debated. It is hypothesized that during systemic infections, there is direct activation of pain-producing mechanisms either by microorganisms or secondary to fever or a combination of both (142), with the subsequent release of proinflammatory substances that play a role in the generation of headache. In local CNS infections such as meningitis and encephalitis, the infective microorganism or its toxins directly invade the meningeal sensory nociceptor terminals causing inflammation and releasing proinflammatory mediators (e.g., bradykinin, prostaglandins [PGDs], and cytokines) (142). The resultant septic meningeal inflammation that causes the headache of meningitis is comparable to the presumed aseptic inflammation of the neurovascular junction of meningeal/dural blood vessels during migraine attacks. Therefore, the phenotypic characteristics of secondary headaches and migraines substantially overlap (142).

#### 2.4.3 Headache in HIV

Human immunodeficiency virus-1 is a neurotropic virus that enters the CNS early and remains latent in glial cells. The infiltration of mononuclear cells triggers the release of cytokines that activate latently infected astrocytes to express the virus (143). Infected macrophages and glial cells can result in toxicity by releasing cytokines such as TNF-α, and IL-1β (144). The stimulation of this inflammatory cascade is closely similar to the pathogenesis of migraine (143). Another proposed mechanism is plasma membrane alterations by HIV-1 itself with the resultant change in intracellular K^+^ and Na^+^ concentrations (143). Depolarization is followed by alterations in ionic gradient and a change of membrane permeability causing increased excitatory signals due to excess glutamate release. In acute neuronal infection with HIV-1, viral proteins such as tat and gp120 were associated with increased production of glutamate excitotoxicity through NMDA receptors stimulation and calcium influx-related excitation (145, 146). Another suggested mechanism involves the release of histamine from mast cells primarily because of viral-mediated cell death (147). This is similar to the pathophysiologic mechanism explained in migraine headaches. Evers et al. suggested that central pain processing structures of the trigemino vascular system may be affected by HIV (148). Secondary headaches in PLWHA may be present with non-specific characteristics depending on the opportunistic infection and the level of immunodepression. Opportunistic infections that cause secondary headaches in PLWHA include: cryptococcal meningitis (39%) and CNS toxoplasmosis (16%) (149). Several ARVs such as zidovudine, efavirenz, amprenavir can cause headaches, though the underlying mechanism is not fully elucidated (150–152).

#### 2.4.4 Mechanism of Headache in Malaria

Headache is one of the most common clinical manifestations of malaria (153). Albeit being a non-specific characteristic, headache accounts for up to 75-80% of clinical manifestations in malaria-infected patients (154, 155) with about 30% of cerebral malaria patients reporting headaches (156). The mechanism of headache in acute malaria is not well understood though excessive cytokine release (such as TNF-α and IL-1β) might be an important factor (157). However, the frequency of headaches in non-cerebral and cerebral malaria is not affected by cytokine levels as cytokine plasma concentrations are not correlated to the severity of malaria (158, 159). Hence, the exact mechanistic pathogenesis of malaria-related headaches requires further studies.

Patients recovering from acute malaria manifest some symptoms known as post-malaria neurologic syndrome (PMNS) even when parasites have been cleared (160), and it seems to be an immune-mediated post-infectious syndrome. However, the precise mechanisms underpinning PMNS development after recovery from severe malaria are not well understood. Headache has been reported in about 10% of PMNS, which is often severe and associated with nausea, profound confusion, and impaired memory (161).

#### 2.4.5 Management of Headache and Future Perspectives

When managing a case of headache, the chosen medication should match the patient’s needs. The choice of treatment is usually guided by the characteristics of the headache attack, such as severity, frequency, disability, associated symptoms, and time-to-peak. Cognizant of the high prevalence of secondary headaches in SSA, etiological diagnosis of the headache is key in ensuring optimal patient management. Acute headache treatment options include acetaminophen and nonsteroidal anti-inflammatory drugs; both inhibit PGDs synthesis and limit subsequent inflammation in the CNS (162). Patients unresponsive to these treatments may require migraine-specific treatments including triptans (serotonin receptor agonists), which block the release of vasoactive peptides that trigger neurogenic inflammation (162). Corticosteroids also decreased headache recurrence, particularly for migraines whose duration exceeded 72 hours (163). A promising novel strategy focuses on CGRP, a potent vasodilator recently incriminated in the pathogenesis of migraine and cluster headaches attacks. Indeed, a randomized trial established the safety and efficacy of CGRP antibodies for the prevention of frequent episodic migraines (164). For certain specific indications (such as ≥4 headaches a month, ≥8 headache days a month, debilitating headaches, and medication-overuse headaches), preventive therapy may be indicated; this usually consists of propranolol or amitriptyline, among other medications (165). Future research should focus on better understanding the various molecular pathways in headache development, as these are crucial for the development of new treatments with specific targets and few side effects. Given the frequency of headache and its burden of disability, the safety, and efficacy of emerging therapies should be assessed in robust trials to provide evidence-based management options.

### 2.5 Sleep Disorders

Sleep disorders are a common problem in SSA with some studies reporting a pooled estimate of prevalence ranging from 16.6-55% in sites in some countries such as Ethiopia, Ghana, Kenya, South Africa, Tanzania, and Uganda (166–169). Sleep disorders have been associated with infectious diseases such as HAT and HIV and involve the activation of the immune system (20, 170, 171).

#### 2.5.1 Sleep: Characterization/Stages and Sleep Disorders

**Figure 3:**
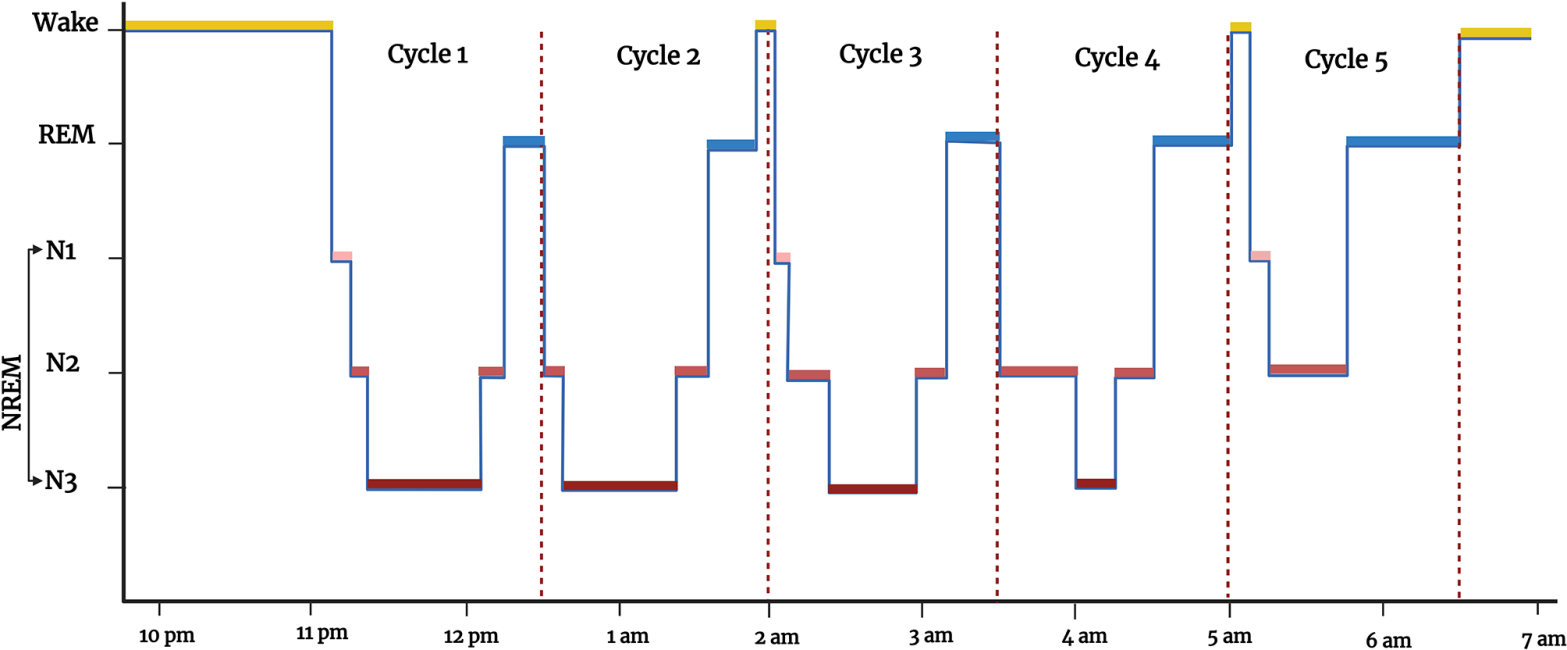
Hypnogram showing the different states of vigilance (wakefulness and sleep) from 10 pm to 7 am: wakefulness (WAKE, in yellow), rapid-eye-movement sleep (REM, in blue), non-rapid eye movement sleep (NREM, in red, which has 3 sub-stages (N1, N2, and N3, in different shades of red). Figure created by EA and WM using BioRender.com.

Sleep is a complex physiologic, recurring, and reversible state of decreased metabolism, responsiveness to external stimuli, and motor activity regulated by a circadian rhythm (172–174). The neurophysiological stages of sleep can be evaluated using polysomnography (PSG), which incorporates EEG for brain electrical activity, electromyogram (EMGs) measuring muscle tone, and electrooculograms (EOGs) that assess eye movement (20, 175). Sleep normally consists of two broad alternating stages: non-rapid eye movement (NREM) sleep and rapid-eye-movement (REM) sleep (172). The NREM is further divided into three stages N1, N2, and N3. From wakefulness, sleep depth increases from N1, N2, N3 to REM, each with distinct neurophysiological characteristics (20). The N1 and N2 are considered light sleep and N3 is also known as slow-wave sleep. A hypnogram is a graph constructed from wakefulness-sleep staging versus time and includes these different stages of sleep, the number of episodes, and their rhythmicity and duration of sleep (**Figure 3**). Actigraphy is another technique to monitor sleep and wakefulness, which is simpler and less expensive than PSG, but only has a binary function (sleep/wake) and does not give details of sleep architecture (20, 176–179).

Chronic disturbances in sleep patterns, poor sleep quality, or sleep-wake disorders are highly prevalent in society and increase with age (180, 181). They adversely affect the quality of life and are associated with significant morbidity and mortality. Sleep disorders can be a symptom of other diseases but can also exacerbate other disorders, especially mental disorders. There are many different types of sleep disorders including insomnia, hypersomnia, parasomnias, narcolepsy, circadian rhythm sleep disorders, sleep apnea, etc. of which insomnia is the most common (180–182).

#### 2.5.2 The Immune System, Systemic Infection, and Sleep

There is a bidirectional relationship between sleep and the immune system (20, 175, 183). Sleep is considered an important restorative and regulatory process for the normal functioning of the immune system (175, 184, 185). Sleep deprivation alters the functioning of immune cells and cytokine expression (175, 185, 186). For example, experimental sleep deprivation reduced natural killer cell activity in humans (187), chronic insomnia decreased the levels of CD3+, CD4+, and CD8+ T cells (188), and sleep deprivation increased the proinflammatory cytokines such as IL-1β, IL-6, and TNF-α (189–191). The immune system also influences sleep. Animal studies have shown that administration of the proinflammatory cytokines IL-1β and TNF-α increase NREM sleep (192), whereas the anti-inflammatory cytokines IL-4 and IL-10 reduce NREM (183). Systemic infections in general cause somnolence as part of sickness behavior, possibly due to the increased levels of inflammatory cytokines and PGDs (20, 175). The increased NREM sleep, reduced REM sleep, and wakefulness during infection are important to preserve energy and support the immune system to fight infections (20, 175, 183).

#### 2.5.3 Human African Trypanosomiasis and Sleep Disorders

Human African trypanosomiasis or sleeping sickness is an endemic disease restricted to Africa where tsetse flies transmit trypanosome parasites to humans. Two subspecies *Trypanosoma brucei (T. b.) gambiense* and *T.b. rhodesiense* cause disease in humans. Another subspecies *T. b. brucei* causes disease in animals and has been used extensively in animal models of the disease. The description of HAT and the parasites that cause it are covered in more detail in the article by Idro et al. in this collection and previous reviews (17).

Human African trypanosomiasis is divided into an early (first) hemolymphatic stage, with general non-specific symptoms of infection, and a late (second) meningoencephalitic stage with neurological and psychiatric manifestations (17, 20). Sleep disturbances are a prominent feature of HAT; thus, it is also known as sleeping sickness (17, 20). They are more pronounced in the late stage of the disease and negatively affect the patient’s quality of life. Sleep disturbances occur in 75% of patients with second-stage HAT caused by *T. b. gambiense* and in 85% of patients with second-stage HAT caused by *T. b. rhodesiense* (193, 194).

Sleep disorders that occur in the second stage of HAT are not the increased sleepiness that normally results from systemic infections, but they are disruptions in sleep structure and circadian rhythm or sleep timing (20, 195). Patients with HAT do not sleep more within a 24-hour period but have fragmented sleep, sleep more during the daytime, and sleep less at night (196, 197). Features of HAT are similar to narcolepsy as patients can fall asleep suddenly (197). Polysomnography studies have shown that HAT patients can move from wakefulness into REM sleep or have very short NREM sleep in between, known as sleep-onset REM periods (SOREM), which is similar to what happens in narcolepsy (197, 198). Actigraphy studies have also shown a disrupted sleep-wake cycle in HAT patients (179) and the actigraphy sleep score has been proposed as a diagnostic and monitoring tool (178).

The sleep disturbances in HAT are caused in part by molecules released from the parasite, such as PGDs, and by the activation of the immune system and inflammation (197). The trypanosomes release PGDs, such as PGD2, and also induce the release of PGD2 from the host, which are somnogenic and can cause disturbances in sleep (197, 199). Activation of the immune system during HAT results in a robust elevation of proinflammatory cytokines and other proinflammatory molecules (17, 200, 201). Cytokines such as TNF-α, IL-1β, and IFN-γ and chemokines such as CXCL10 are upregulated in the brain and CSF of animal models of HAT (17, 201–204) and the CSF of second-stage HAT patients (202, 205). These cytokines such as TNF-α, IL-1β, and IFN-γ alter sleep patterns and circadian rhythm (197, 206). The levels of immune molecules and PSG correlate to the actigraphy findings (178). The levels of cytokines and chemokines have also been suggested as biomarkers for staging HAT (202, 205) and could be possible biomarkers to monitor therapeutic outcomes in HAT patients. More studies are needed to produce diagnostic kits or tools to monitor these molecules to stage the disease and monitor therapeutic outcomes.

#### 2.5.4 Human Immunodeficiency Virus and Sleep Disorders

##### 2.5.4.1 Prevalence of Sleep Disorders and Nature of Sleep Disorders Among PLWHA

A high percentage of PLWHA suffer from poor sleep quality, with an overall prevalence of 58% (207). In some studies done in the SSA region, the prevalence of poor sleep quality in PLWHA ranged from 57% to 61% (171, 208, 209). The sleep disturbances experienced by PLWHA include hypersomnia, insomnia, difficulties in initiating sleep (longer sleep onset latency), fragmented sleep, sleep apnea, and restless leg syndrome, with variable changes in the NREM and REM sleep, as well as circadian rhythm disorders (208, 210–216).

##### 2.5.4.2 Causes of Sleep Disorders Among PLWHA

The pathogenesis of sleep disorders in PLWHA is multifactorial and includes the ARVs, effects of the immune system and viral molecules, disease progression, opportunistic infections, substance abuse, depression, and financial and social concerns (20, 171, 208). Some ARVs especially the non-nucleoside reverse transcriptase inhibitors (NNRTIs) such as efavirenz cause sleep disturbances such as insomnia, somnolence, and nightmares (189, 208, 217).

Sleep disorders are comorbid with various disorders such as anxiety, depression, and pain in PLWHA (171, 180, 189, 211, 218). There is a bidirectional relationship between sleep disorders and other disorders such as anxiety, depression, and pain. Sleep disorders contribute to the development of anxiety, depression, and pain, and on the other hand anxiety, depression, and pain cause and worsen sleep disorders (20, 219–221).

##### 2.5.4.3 The Immune System and Sleep Disorders Among PLWHA

Given the bidirectional relationship between sleep and the immune system and the alterations in the immune system caused by HIV infection, it is plausible to hypothesize that alterations in the immune system contribute to sleep disturbances in PLWHA and vice versa.

Various studies have investigated the relationship between sleep disturbances and immune system activation in PLWHA with non-conclusive results (171, 222–227). In a study in South Africa on PLWHA taking ART for 4 years, poor sleep quality correlated with both higher current CD4+ cell count and more upregulation of CD4+ cells from baseline (before taking ART) (171). Most of the patients in this cohort started ART late and this has been associated with spontaneous immune activation because of the increase of CD4+ cell count (171, 228), which would lead to an inflammatory state. This is in contrast with some earlier studies that showed a correlation between low CD4+ cell count and poor sleep quality (209, 229, 230). Recently, long sleep hours were associated with low CD4+ cell count and greater severity of the disease (210).

A recent study conducted in the United States of America (USA) did not find a significant association between insomnia and monocyte activation marker soluble CD14 (sCD14) or the proinflammatory cytokine IL-6 (227). In HIV-positive men who have not received ART, high TNF-α concentrations were associated with moderate-to-severe obstructive sleep apnea independent of CD4+ cell count and plasma HIV-RNA concentration (222). Sleep onset insomnia was associated with single nucleotide polymorphisms (SNPs) for IL-1β, IL-6, IL13, and TNF-α in PLWHA classified as having sleep onset insomnia (Gay et al., 2014). However, plasma levels of the proinflammatory cytokines IL-1β, IL-2, IL-6, IL-10, IL-13, and TNF-α did not differ between those with sleep onset insomnia and those without (223). A higher percentage of wake after sleep onset (WASO%) was associated with SNPs of IL1R2 and TNF-α, whereas SNPs of IL-2 were associated with less WASO%. Single nucleotide polymorphisms of IL-1R2 and TNF-α were also associated with short sleep duration (224). Higher levels of c-reactive protein (CRP) and IL-6 in PLWHA were associated with disturbances of various sleep metrics such as later sleep onset, lower total sleep time, and higher WASO (226). Moore et al. reported sex-dependent differences in cytokines and sleep disturbances in PLWHA (225). They observed significant negative correlations between sleep disturbance and the proinflammatory cytokines IFN-γ and TNF-α, but not IL-6, in females, with no significant associations among males.

Studies that have been done in the USA reported an association between inflammatory cytokine levels or polymorphisms and sleep disturbances (222–224, 226). Thus, there is a need for such studies in the SSA region, which has the highest number of PLWHA and has a peculiar situation including PLWHA who started ART later than those in the USA. Further studies are also needed to ascertain whether immune molecules can be used as biomarkers of sleep disorders and to measure the response to interventions to alleviate sleep disorders and comorbid conditions such as anxiety, depression, and pain. The study by Moore et al. that showed sex-dependent differences in inflammatory molecules and sleep disturbances in PLWHA, suggests that this could be the reason for the variability amongst various studies, hence further studies are needed (225).

### 2.6 Peripheral Neuropathy and Neuropathic Pain

Peripheral neuropathies (PN) and disorders of the peripheral nervous system are common problems caused by various acquired conditions such as diabetes mellitus, chemotherapy, HIV and other infectious diseases, alcoholism, nutrient deficiencies, or toxic molecules. Furthermore, inherited conditions such as Charcot-Marie-Tooth and Fabry disease represent less frequent etiologies of PN (10, 231, 232). Patients present with predominant sensory symptoms (numbness, tingling, burning, stabbing, or electrical pain), motor symptoms (muscle weakness, wasting, twitching and cramps and paralysis), and autonomic symptoms (orthostatic hypotension, sweat abnormalities, gastroparesis, esophageal dysfunction, bladder dysfunction) (232, 233). Peripheral neuropathies have been reported to affect about 15% of adults in the USA, with diabetics having a higher prevalence of PN (234). In a study conducted in urban and rural Uganda, neurological disorders’ overall point prevalence was 3.3%, of which the majority was due to PN, with a crude prevalence of 33.7% (3). In another study conducted in rural Uganda, PN was present in 13% of the cohort and was more common in HIV-positive participants (235).

#### 2.6.1 Peripheral Neuropathy Related to Infectious Diseases

Infectious causes of neuropathy include HIV, hepatitis viruses, varicella-zoster virus, herpes simplex viruses, flaviviruses, rabies virus, human T-cell lymphotrophic virus type-1, *Mycobacterium leprae*, *Borrelia burgdorferi*, *Corynebacterium diphtheria*, *Clostridium botulinum*, and *Trypanosoma cruzi* (10, 236–240). Hepatitis B, C, D are all associated with several forms of neuropathies, and hepatitis A is also associated with a rare form of neuropathy (236). This is of specific concern to the African region as viral hepatitis is considered an endemic public health problem (241). Similarly, the rabies virus has a higher prevalence in Africa and Asia with 95% of rabies-related deaths occurring in these areas (236, 242). Although the prevalence of leprosy, caused by *Mycobacterium leprae*, has been largely decreasing, neuropathy is the main manifestation associated with it (236, 237) and leprosy is the major cause of neuropathy in some endemic countries in the SSA such as Ethiopia (10). Globally the number of new cases of leprosy detected annually is around 200,000, with Africa contributing around 20,000 cases (243). Peripheral neuropathy is an integral part of leprosy and thus is briefly described below. According to the Joint United Nations Programme on HIV/AIDS (UNAIDS) 2021, preliminary epidemiological estimates show that 36.7 million people are living with HIV, of which 25.3 (68.9%) millions are in the SSA region (244). Since HIV has the highest prevalence in SSA and has a broad range of associated neuropathies, it is discussed in more detail below.

#### 2.6.2 Leprosy

Leprosy causes irreversible nerve damage, and peripheral neuropathy is present in all forms of leprosy. The sensory neuropathies of leprosy present in various forms including cutaneous nerve damage (resulting in anesthetic or hypo-aesthetic skin lesions), symmetrical pansensory neuropathy, and leprous ganglionitis (245). Both the innate and adaptive immune systems are involved in nerve damage during leprosy (246). *Mycobacterium leprae* invades and/or activates immune cells such as macrophages and T cells as well as Schwann cells, which contribute to the nerve damage that occurs during leprosy (245–247). The bacteria also infect the nerves and cause an inflammatory process that leads to the damage and the thickening of nerves in about 40 to 75% of infected individuals, which is painful most of the time (245). Depending on the type of lesions, there is either Th1 or Th2 immune response plus CD8 cell involvement (245, 246). A Th1 cytokine response (IFN-γ, IL-2, IL-15, TNF-α) is associated with tuberculoid leprosy lesions, while a Th2 cytokine response (IL-4 and IL-10) is associated with lepromatous leprosy lesions (245, 246). Activated T cells attack and kill Schwann cells, which then affects nerve cell function. Infected macrophages cause axonal damage and demyelination through increased production of nitric oxide (NO) and reactive nitrogen species (247).

#### 2.6.3 Human Immunodeficiency Virus-Associated Neuropathy and Neuropathic Pain

Human immunodeficiency virus-associated neuropathy is one of the main causes of neuropathies in SSA due to the high prevalence of HIV in the region (248). The prevalence of PN in PLWHA in several SSA countries ranges from 18-52% (235, 249–251). This neuropathy is caused by both the virus and ART (236, 237). The most common form of neuropathy is distal symmetrical polyneuropathy (DSP) with almost one-third of HIV patients facing this complication (236). Distal symmetrical polyneuropathy is associated with advanced stages of HIV disease and develops as immunosuppression progresses and as HIV viral load increases (252). The DSP caused by some ARVs such as the nucleoside reverse transcriptase inhibitors (NRTIs) is called antiretroviral toxic neuropathies (ATN) and is clinically indistinguishable from HIV-DSP, but they have different pathophysiological mechanisms (236). Other drugs commonly used in HIV-associated infections that may cause DSP include isoniazid, ethambutol, and dapsone (253). Distal symmetrical polyneuropathy may be detected pathologically in nearly all patients dying with AIDS (254). Symptoms of DSP include burning feet, numbness, and paresthesias (255). However, in some patients DSP is asymptomatic. Signs of motor involvement are seen in very few patients until the very late stages of DSP.

Another type of neuropathy associated with HIV that affects African patients is diffuse infiltrative lymphocytosis syndrome (236). In addition, cytomegaloviruses, opportunistic infections, and necrotizing vasculitis during advanced HIV can cause severe mononeuropathies (236). Other forms of neuropathies related to HIV include inflammatory neuropathies and radiculopathies (256).

The prevalence of neuropathic pain in PLWHA is 35%, due to the virus and medications used to treat it (257, 258). Neuropathic pain is defined by the International Association for the Study of Pain (IASP) as ‘Pain caused by a lesion or disease of the somatosensory nervous system’ (259). Symptoms include both negative (hypoesthesia, hypoalgesia, numbness, loss of sensation) and positive sensory symptoms (hyperalgesia, evoked pain, spontaneous pain). The pain is progressive, starts in the feet and ascends symmetrically to the hands, and is described as “glove and stocking” distribution (256, 260).

Although HIV-associated neuropathic pain negatively affects the patient’s quality of life, to date there are no approved FDA medications to either prevent it or treat it (261, 262). In the case of ATN, substituting the offending drug is the first step in treatment, which may still be challenging in some parts of Africa due to limited access to drugs due to procurement difficulties even though more antiretroviral drug options have become available in recent years. Some drugs used for other types of neuropathic pain such as anticonvulsants, antidepressants, topical agents, as well as non-steroidal anti-inflammatory drugs, and opioids are used and show modest activity (261, 263). However, in clinical trials, antidepressants such as amitriptyline (264, 265), and anticonvulsants such as pregabalin (266) were not effective for the management of HIV-DSP. In a multisite study, PLWHA rated the overall effectiveness of self-care pain management strategies on a scale of 1 to 10 as follows: reflexology (7.53), meditation (7.08), prescribed antiepileptics (6.85) massage (6.84), marijuana (6.82), acupuncture (6.81), feet elevation (6.53) and taking a hot bath (6.45) (267, 268). Those numbers reflect that both medications and self-care management strategies provide inadequate pain management. Thus, there is a need to find new drugs to prevent or alleviate HIV-associated neuropathic pain. Understanding the pathophysiological mechanism of HIV-DSP and the involvement of the immune system may provide new therapeutic targets to manage it.

#### 2.6.4 Human Immunodeficiency Virus-Associated Neuropathy, Neuropathic Pain, and the Immune System

**Figure 4:**
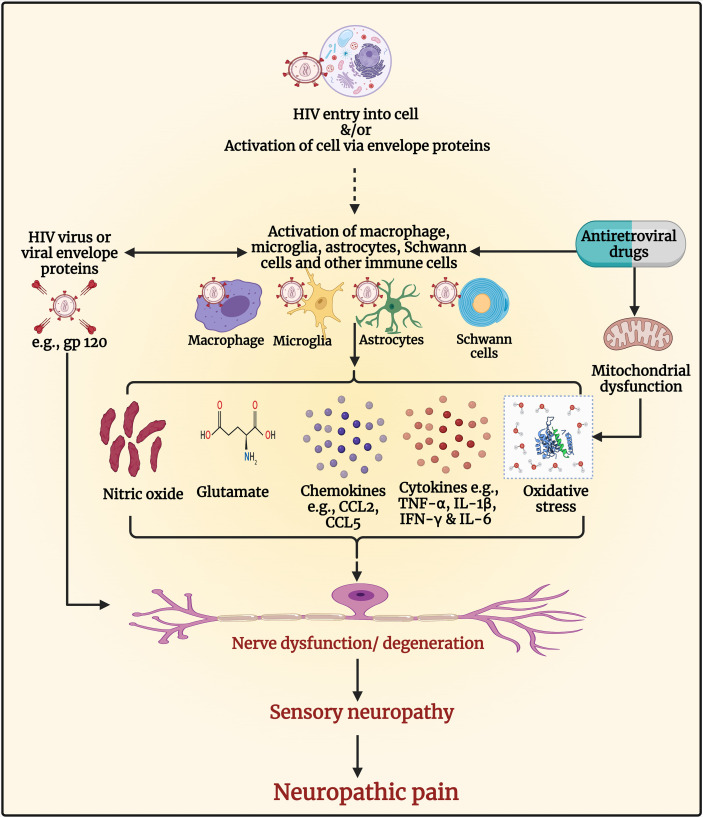
Pathogenesis of human immunodeficiency virus (HIV) associated sensory neuropathy and neuropathic pain. The entry of HIV in macrophages or microglia results in their activation and the release of proinflammatory cytokines, chemokines, glutamate, nitric oxide, and viral envelope proteins, including glycoprotein (gp)120, which can cause nerve dysfunction/neurodegeneration. The viral envelope gp120 has a direct neuropathic effect on neurons due to activation of chemokine receptors resulting in neuronal hyperexcitability and neuropathic pain. It also has indirect neuropathic effects through the activation of macrophages and Schwann cells. The presence of proinflammatory cytokines within the peripheral nerve and dorsal root ganglia causes infiltration of macrophages and lymphocytes, which secrete inflammatory cytokines (e.g., tumor necrosis factor-alpha [TNF-α], interleukin-1 beta [IL-1β], interferon-gamma [IFN-γ], and IL-6), and chemokines (e.g., C-C motif ligand 2 [CCL2] and CCL5) and exacerbate nerve degeneration leading to neuropathy and neuropathic pain. Antiretroviral drugs such as nucleoside reverse transcriptase inhibitors (NRTIs) inhibit deoxyribonucleic acid (DNA) γ- polymerase, the enzyme essential for copying and repair of mitochondrial DNA. This results in the accumulation of mutations of mitochondrial DNA, defective respiratory chain subunits, impaired oxidative phosphorylation, reduced adenosine triphosphate (ATP), and oxidative stress. Oxidative stress causes nerve degeneration. The NRTIs also contribute to neuropathy and neuropathic pain by activating glial and immune cells to release cytokines, chemokines, and molecules that induce neuronal hyperexcitability and neurodegeneration. Figure created by EA and WM using BioRender.com.

Various mechanisms are involved in the development of HIV-DSP and neuropathic pain (see **Figure 4**). Products of immune activation in response to HIV infection, along with HIV proteins are involved (269). The entry of HIV in macrophages or microglia results in their activation and the release of proinflammatory cytokines, chemokines, glutamate, and viral envelope proteins, including gp120. The viral envelope gp120 that HIV uses to interact with the CD4 receptors and enter the cells, has a direct neuropathic effect on neurons due to activation of chemokine receptors or indirectly through activation of macrophages and Schwann cells (25). In PLWHA, the presence of these proinflammatory cytokines causes infiltration of macrophages and lymphocytes within the peripheral nerve and dorsal root ganglia (DRG) (270–275). The infiltrating macrophages and lymphocytes secrete inflammatory cytokines (TNF-α, IL-1β, IFN-γ, and IL-6) and chemokines and exacerbate nerve degeneration leading to the loss of the small unmyelinated sensory fibers followed by the large myelinated fibers in a dying back pattern of nerve degeneration (260, 276–278). Several chemokine receptors, including C-X-C chemokine receptor type 4 (CXCR4) and C-C chemokine receptor type 5 (CCR5), are expressed widely in the nervous system, for instance in the DRG satellite glial cells. The binding of the viral gp120 with CXCR4 receptors enhances the production of the chemokine C-C motif ligand 5 (CCL5) also known as regulated upon activation, normal T cell expressed and secreted (RANTES) chemokine which then binds to CCR5 receptors and enhances the release of TNF-α, which may induce neurotoxicity and cause axonal degeneration (269, 279, 280). Increased activation of CXCR4 receptors by chemokines, HIV gp120 or NMDA receptors by glutamate, increases calcium influx and stimulates downstream signaling cascades and subsequent production of second messengers particularly kinases, including protein kinase A, protein kinase C, mitogen-activated protein kinase (MAPK), and phosphoinositide 3-kinase (281–283).

The increase of calcium levels inside the neuron facilitates nitric oxide synthase (284) to produce NO which further enhances pain *via* the generation of proinflammatory cytokines (285). Mounting evidence has shown that free radicals are involved in causing pain (286–291). In addition, gp120 activates microglia and astrocytes, which upregulate ROS that disrupts mitochondrial transmembrane potential (25, 260, 277, 292). The ROS produced by the interaction of viral gp120 with the receptors present either on microglia or neuron modulates apoptosis through TNF-α and its receptors (293); all these molecules have known neurotoxic properties and may be associated with axon degeneration, neuroinflammation, and hyperalgesia (294, 295).

Mitochondrial toxicity is the main mechanism responsible for ATN (296–299). The increased superoxide levels in patients with ongoing HIV infection damages both neurons and astrocytes and causes neuroinflammation (295, 300). Administration of ddC (a highly neurotoxic out of clinical use NRTI) causes neuropathy *via* several mechanisms including immune system activation. Treatment of rats with ddC, resulted in increased levels of both transcripts and protein levels of TNF-α in the spinal cord and DRG neurons, at a time point when the rats had developed mechanical allodynia, a symptom of neuropathic pain (301, 302). Aging mice treated with ddC had increased neuroinflammation as microglia and astrocytes were activated, and TNF-α, IL-1β, and Wnt5a were upregulated, in the spinal cord (303). Treatment of mice with other NRTIs (zidovudine, lamivudine, stavudine) up-regulated cytokines, including IL-1β, TNF-α, and IL-6 in different brain regions (304). Elevated CCL2 in DRG accompanied by a reduction in intraepidermal nerve fiber density and spinal gliosis have been observed in a model for HIV-sensory neuropathy and ATN using gp120 and ddC (305). In a recent study using female mice, systemic ddC administration induced transcript levels of cytokines (IL-1β, IFN-γ, and TNF-α) in the brain and paw skin, and the phosphorylation levels of the signaling molecule Erk1/2 in the brain, which was associated with the development of mechanical allodynia (306). These effects of NRTIs can augment the effects of HIV, as the virus activates p38 MAPK, Erk1/2 pathways to aid in its replication and proliferation, which is harmful to the host cell as this leads to the release of proinflammatory cytokines and biomarkers that signal apoptosis (307). These studies suggest that proinflammatory cytokines both in the periphery and in the CNS play a role in the pathophysiology of ATN.

Besides NRTIs, other ARVs such as protease inhibitors (PIs) can cause ATN. The PIs such as indinavir, saquinavir, or ritonavir have been reported to cause sensory PN in PLWHA (308). *In vitro*, indinavir caused neuronal atrophy and DRG macrophage cytotoxicity (308). Administration of indinavir induced mechanical allodynia in rats, which was associated with increased expression of phospho-p38 in microglia (309).

#### 2.6.5 Value of Neuroimmune Changes in Therapeutics

Immunomodulators that reduce the expression of the inflammatory cytokines and or inhibit their signaling pathways could be of therapeutic use in the prevention and management of neuropathic pain in PLWHA. B-caryophyllene (BCP), a cannabinoid type 2 receptor (CB2R)-selective phytocannabinoid, prevented the development of and attenuated ddC-induced allodynia and the expression of proinflammatory cytokines and the signaling molecule, Erk1/2 (306). Other immunomodulators such as minocycline and pentoxifylline also prevented the development of ddC-induced allodynia (302, 306) and alleviated established ddC-induced hyperalgesia and allodynia (310). Administration of IL-10 reduced mechanical allodynia and reversed the upregulation of p-p38 MAPK, TNF-α, SDF-1α, and CXCR4 in a model of gp120 and ddC induced HIV-sensory neuropathy and ATN (311). These animal studies warrant further research to evaluate if they can be translated to therapeutic drugs in PLWHA suffering from neuropathic pain.

## 3 Concluding Remarks

Neuroinfections prevalent in the SSA region cause various neurological disorders such as epilepsy, dementia, motor neuron diseases, headache, sleep disorders, and peripheral neuropathy. Infections provide an excellent opportunity to understand the pathophysiology of many primary neurological disorders, since they may give valuable clues about the real reason of the disorder including molecules/pathways or structural damages involved in these disorders. The immune system plays an important role in the pathophysiology of these neurological disorders.

### 3.1 Epilepsy

Epilepsy hugely affects SSA, with CNS infections as the most frequent preventable cause. Epilepsy often occurs after an initial brain insult followed by a latent phase during which an enduring epileptogenic lesion is established in the patient’s brain. Although the pathophysiological mechanisms are not fully understood, CNS infection and neuroinflammation result in the release of pro-inflammatory cytokines by glial cells and neurons. Over time, the inflammatory cascade leads to neuronal loss, gliosis, and NMDA/glutamate-mediated brain hyper-excitability underpinning chronic epileptogenesis. More research is warranted to understand the risk factors, mechanisms, and specific triggers for the development of epilepsy following a CNS infection. This will eventually pave the way for better preventive and therapeutic approaches for epilepsy in SSA.

### 3.2 Dementia

With the increase of the aging population in SSA, the number of persons with neurodegenerative diseases is expected to rise over time. The pathophysiological processes underpinning the development of dementia include chronic neuroinflammation that activates microglia to release cytokines and neurotoxic substances. The hypothesized development of AD *via* a seeding mechanism is an elegant illustration of how CNS invasion by microbes could increase the risk for non-communicable neurodegenerative conditions. Furthermore, the role of peripheral inflammation in fostering CNS inflammation remains an interesting research direction that could open new therapeutic avenues for dementia and other neurodegenerative conditions.

### 3.3 Motor Neuron Diseases

Research on MNDs occurrence has identified an interaction between genetic, age-related, environmental, and developmental factors. An underlying neuroinflammatory process consisting of activated microglia, infiltrated T cells, and the subsequent overproduction of pro-inflammatory cytokines constitute a pathological hallmark of MND. These have been documented in individuals infected by viruses (poliovirus, HIV) or activation of endogenous retroviruses such as HERV-K, and often present as ALS-like syndrome. There is currently no cure for ALS. Further understanding of molecular pathology within glial cells will contribute to developing therapeutics that will slow the progression of MNDs and reduce incidence as well as disabilities.

### 3.4 Headache

Headaches have a huge burden worldwide and neuroimmunology plays a key role in the pathogenesis. The immunopathologic mechanisms underlying headache, both primary and secondary, are non-specific. They involve an interplay of pro- and anti-inflammatory cytokines, stimulating brain dural nociceptors. While there has been considerable advancement in our understanding of neuroimmunology, the mechanisms underlying the genesis of headache during systemic infections are still speculative, ranging from direct (pathogen-related) to indirect (drug-induced and post-infectious) influence. There is a need for further exploration to fill knowledge gaps, including the triggering factors and the exact immune-mediated mechanisms involved in both primary and secondary headaches, to achieve better management strategies in the future.

### 3.5 Sleep Disorders

The immune system and more specifically proinflammatory cytokines contribute to the pathogenesis of sleep disorders during infectious diseases such as HAT and HIV. How and why the cytokines such as IFN-γ and TNF-α contribute to sleep disturbances in PLWHA in a sex-dependent manner needs to be elucidated as well as the contribution of cytokine polymorphisms to sleep disturbances in PLWHA. Inflammatory cytokines such as TNF-α, IL-1β, and IFN-γ may alter sleep patterns and the circadian rhythm during HAT. More studies on how these molecules contribute to the alteration of sleep patterns during HAT are needed. These cytokines and chemokines such as IFN-γ-induced CXCL10 seem to be useful biomarkers for staging HAT.

### 3.6 Peripheral Neuropathy and Neuropathic Pain

Both the HIV and the ARVs used to treat the virus cause sensory neuropathy and neuropathic pain. They both activate glial and immune cells to release proinflammatory cytokines such as TNF-α, IL-1β, IFN-γ, and IL-6, and chemokines such CCL2 and CCL5, which cause neuronal hyperexcitability, neurodegeneration, neuropathies including neuropathic pain. Animal studies suggest that immunomodulatory drugs that inhibit the expression or secretion of these proinflammatory cytokines could prevent or alleviate HIV-associated neuropathy and pain. Of interest are the cannabinoids, taking into consideration that some clinical trials have shown that smoked cannabis alleviates neuropathic pain in PLWHA. However, the use of cannabis is limited by its psychoactive side effects, which are CB1R-dependent. Animal studies showing that the non-psychoactive CB2R agonists alleviate NRTI-induced allodynia and inhibit the expression of proinflammatory cytokines suggest that these molecules could be useful for the management of neuropathic pain in PLWHA with a better side effect profile.

In conclusion, the immune system plays an important role in the pathogenesis of neurological disorders caused by neuroinfections. Further understanding of the role of the immune system in the pathogenesis of these neurological disorders during neuroinfections is vital for the development of therapeutics as well as biomarkers for diagnosis and therapeutic monitoring of these disorders.

## Author Contributions

WM participated in the conception of the article idea, which was discussed by all authors before writing began. LN, JNSF, EA, WM, AKN participated in the writing different sections of the article. EA put together all the different sections of the article, did the final formatting of the article, and all authors critically reviewed and edited the manuscript. All authors contributed to the article and approved the submitted version.

## Funding

EA was supported by a research grant from Kuwait University, Research Sector (YP03/18). WM was supported by grants from Kuwait University, Research Sector (YP03/18 and PT02/15) and some of the studies described in this review have been supported by grants from the US NIH/Fogarty (1R21NS064888-01A1), from the Wellcome Trust (WT089992MA) and the Swedish Research Council (04480). AKN received support from the following research grants: the European Commission (NEUROTRYP Grant FP6-2004-INCO-DEV-3 032324); European Research Council (ERC Grants Nos. 671055, 768815), the US NIH/Fogarty (1R21NS064888-01A1), and the Wellcome Trust (WT089992/Z/09/Z) for some of the studies described in this review.

## Conflict of Interest

The authors declare that the research was conducted in the absence of any commercial or financial relationships that could be construed as a potential conflict of interest.

## Publisher’s Note

All claims expressed in this article are solely those of the authors and do not necessarily represent those of their affiliated organizations, or those of the publisher, the editors and the reviewers. Any product that may be evaluated in this article, or claim that may be made by its manufacturer, is not guaranteed or endorsed by the publisher.
